# Modulation of long-term potentiation-like cortical plasticity in the healthy brain with low frequency-pulsed electromagnetic fields

**DOI:** 10.1186/s12868-018-0434-z

**Published:** 2018-06-13

**Authors:** Enrico Premi, Alberto Benussi, Antonio La Gatta, Stefano Visconti, Angelo Costa, Nicola Gilberti, Valentina Cantoni, Alessandro Padovani, Barbara Borroni, Mauro Magoni

**Affiliations:** 1 Stroke Unit, Azienda Socio Sanitaria Territoriale “Spedali Civili”, “Spedali Civili” Hospital, Piazza Spedali Civili 1, 25123 Brescia, Italy; 2 Neurology Unit, Department of Clinical and Experimental Sciences, University of Brescia, Brescia, Italy; 3 cNVR Consorzio Veneto di Ricerca, Padua, Italy; 4 Rehabilitation Unit, Casa di Cura “Villa Barbarano”, Salò, Brescia Italy

**Keywords:** Long-term potentiation-like cortical plasticity, Low frequency-pulsed electromagnetic fields, Diamagnetism, Neuroplasticity

## Abstract

**Background:**

Non-depolarizing magnetic fields, like low frequency-pulsed electromagnetic fields (LF-PEMFs) have shown the ability to modulate living structures, principally by influencing synaptic activity and ion channels on cellular membranes. Recently, the CTU Mega 20 device was presented as a molecular accelerator, using energy up to 200 J and providing high-power (2 Tesla) pulsating fields with a water-repulsive (diamagnetic) action and tissue biostimulation. We tested the hypothesis that LF-PEMFs could modulate long-term corticospinal excitability in healthy brains by applying CTU Mega 20^®^. Ten healthy subjects without known neurological and/or psychiatric diseases entered the study. A randomized double-blind sham-controlled crossover design was employed, recording TMS parameters (amplitude variation of the motor evoked potential as index of cortical excitability perturbations of the motor system) before (pre) and after (post + 0, + 15, + 30 min) a single CTU Mega 20 session on the corresponding primary right-hand motor area, using a real (magnetic field = 2 Tesla; intensity = 90 J; impulse frequency = 7 Hz; duration = 15 min) or sham device. A two-way repeated measures ANOVA with TIME (pre, post + 0, + 15, + 30 min) and TREATMENT (real vs. sham stimulation) as within-subjects factor was applied.

**Results:**

A significant TIME × TREATMENT interaction was found (*p* < 0.001). Post hoc comparisons showed a significant effect of TIME, with significant differences at + 0, + 15 and + 30 min compared to baseline after real stimulation (all *p* < 0.05) but not after sham stimulation (all *p* < 0.05) and significant effects of TREATMENT, with significant differences at + 0, + 15 and + 30 min for real stimulation compared to sham stimulation (all *p* < 0.005). No significant depolarizing effects were detected throughout the (real) stimulation.

**Conclusions:**

Our proof-of-concept study in healthy subjects supports the idea that non-ionizing LF-PEMFs induced by the CTU Mega 20 diamagnetic acceleration system could represent a new approach for brain neuromodulation. Further studies to optimize protocol parameters for different neurological and psychiatric conditions are warranted.

*Trial Registration* The present work has been retrospectively registered as clinical trial on ClinicalTrials.gov NCT03537469 and publicly released on May 24, 2018

**Electronic supplementary material:**

The online version of this article (10.1186/s12868-018-0434-z) contains supplementary material, which is available to authorized users.

## Background

Several studies have investigated the effects of different stimulation methods in modulating human brain plasticity [1–3]. Among these, the paired associative stimulation (PAS) paradigms were shown to modulate the excitability of corticospinal fibers related to the primary motor cortex, as a form of long-term modulation, including long-term potentiation (LTP) or depression (LTD) linked to synaptic plasticity [4, 5]. All these approaches induce electric currents to obtain a depolarization in the stimulated brain regions [6, 7]. However, even non-depolarizing magnetic fields, as in static magnets [8–10] or Low Frequency-Pulsed Electromagnetic Fields (LF-PEMFs) [11, 12], have shown the potential to modulate living structures [8, 13]. In particular, these non-depolarizing approaches seem to influence synaptic activity and ion channels on cellular membranes [8]. Indeed, it has been suggested that LF-PEMF can influence numerous types of changes in cells including migration, cell differentiation, stress response, potentially affecting morphology, migration of embryonic cells, and cell reprogramming [13–17]. Furthermore, it has been reported that LF-PEMF promotes osteogenic and neurogenic differentiation, which has been clinically used to repair bone fractures, promote wound healing [18, 19], and has been shown to have a neuroprotective effect after ischemic stroke in mice during the recovery process [11]. Several trials have also assessed the effects of LF-PEMF on major depressive disorder and unipolar or bipolar depression [20–22]. Moreover, LF-PEMF has been reported to influence brain glucose metabolism, thus affecting local brain activity [23]. Collectively, these studies indicate that LF-PEMF may be involved in neuroprotection.

**Fig. 1:**
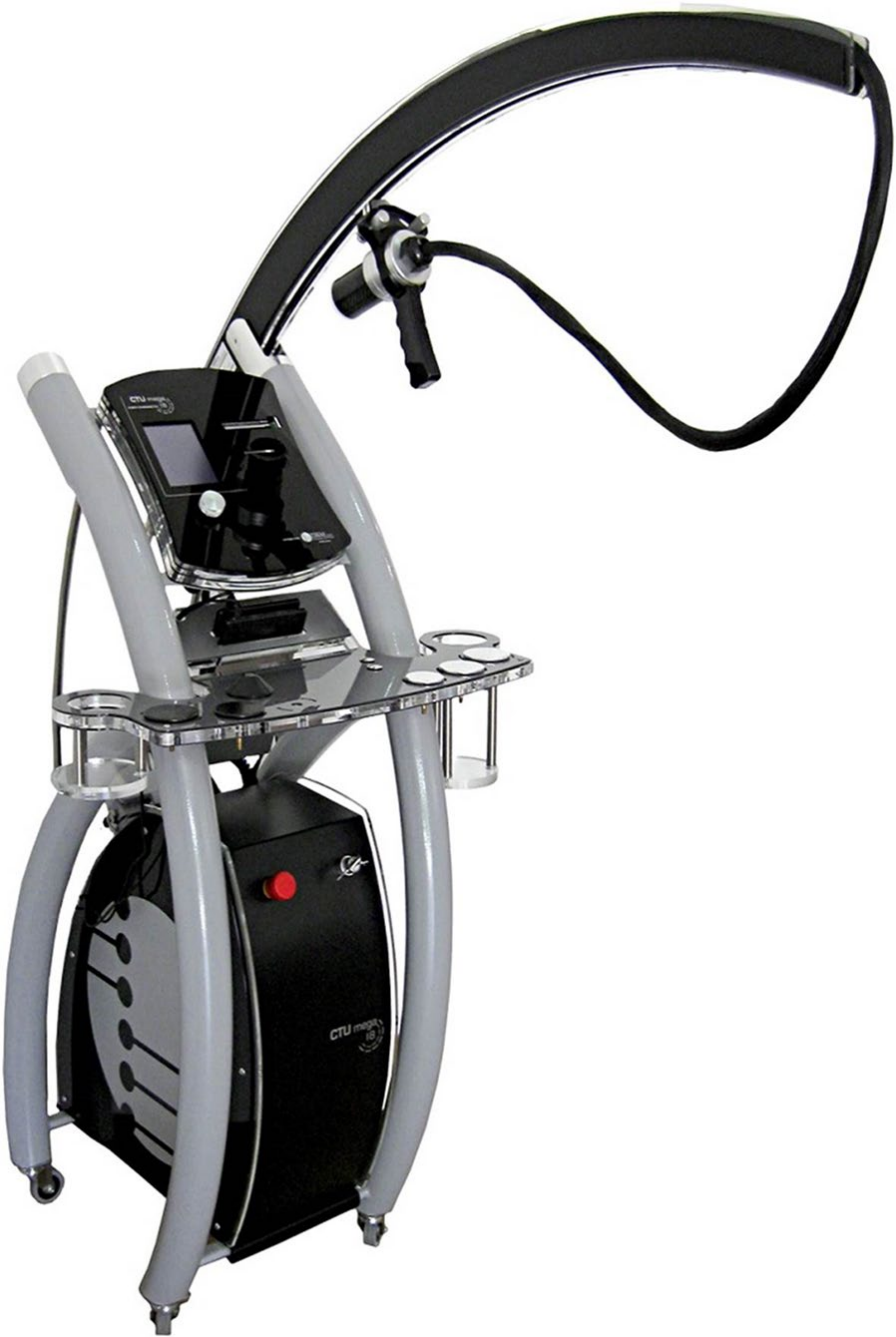
CTU Mega 20 device. The original equipment used in the study, directly provided by PERISO SA (http://www.periso.ch/)

LF-PEMFs (< 50 Hz) can be considered as a class of non-ionizing radiation with an associate energy < 12 electronvolt (eV), not enough to induce ionization phenomena [24], but with potential effects on biological components [25]. Recently, the CTU Mega 20^®^ device (see Fig. 1) was presented as a molecular accelerator, using an energy up to 200 J and providing high-power (2 Tesla) pulsating fields with a water-repulsive (diamagnetic) action with a consequent tissue biostimulation [25, 26]. In this research we tested the hypothesis that LF-PEMFs could modulate long-term corticospinal excitability in the healthy brain by applying transcranial pulsed magnetic fields with CTU Mega 20^®^ (http://www.periso.ch/). Therefore, we employed single-pulse transcranial magnetic stimulation (TMS), which allows an in vivo registration of the amplitude variation of the motor evoked potentials as a tool to explore cortical excitability perturbations of the motor system after CTU Mega 20 application.

## Methods

### Subjects

Ten healthy subjects without known neurological or psychiatric diseases were recruited for this study (mean age ± standard deviation: 25.5 ± 3.8 years) (see Table 1).

**Table 1:** Demographic characteristics and neurophysiological parameters

Variable	Real (n = 10)	Sham (n = 10)	*p*
Age (mean ± SD)	25.5 ± 3.8	25.5 ± 3.8	–
Gender % (no female)	50 (5)	50 (5)	–
Educational level, years	21.5 ± 1.9	21.5 ± 1.9	–
Handedness % (no right)	100 (10)	100 (10)	–
Correct hypothesis on treatment %	50	50	–
rMT (% of MSO)	46.4 ± 6.5	46.2 ± 6.5	n.s.
Corticospinal excitability baseline (mV)	1.01 ± 0.12	1.05 ± 0.05	n.s.
Corticospinal excitability post + 0 (mV)	1.60 ± 0.19	1.10 ± 0.14	*p* = 0.001
Corticospinal excitability post + 15 (mV)	1.69 ± 0.18	1.12 ± 0.21	*p* < 0.001
Corticospinal excitability, post + 30 (mV)	1.57 ± 0.34	1.07 ± 0.20	*p* = 0.002

*MSO* max stimulator output, *SD* standard deviation, *mV* millivolt

The subjects and operators who performed TMS were blinded to the type of stimulation applied.

Written informed consent was obtained from all subjects according to the Declaration of Helsinki. The study protocol was approved by the local ethics committee. This study adheres to CONSORT guidelines (http://www.consort-statement.org/) (see Additional file 1 for CONSORT checklist).

### CTU Mega 20 stimulation

The CTU Mega 20 diamagnetic acceleration system discharges high-field magnetic impulses (with a duration of 5 ms and a period of 1000 ms), generating a magnetic field up to 2 Tesla, with a frequency of 7500 Hz in a volume of approximately 27 cm^3^ [12]. See Additional file 2 for a detailed technical description of CTU Mega 20 physics principles and function.

To assess the effect of LF-PEMFs provided by CTU Mega 20 we employed a randomized double-blind sham-controlled crossover design, recording TMS parameters before (pre) and after (post + 0, + 15, + 30 min) a single-session of CTU Mega 20 on the corresponding primary right-hand motor area, using a real (magnetic field = 2 Tesla; intensity = 90 J; frequency of impulses = 7 Hz; duration = 15 min) or the sham device. Subjects were randomly assessed for real or sham protocol stimulation, in a 1:1 ratio, with a mean interval of 16.9 ± 2.1 days between sessions.

To detect differences in the perception of the stimulation, we asked the patients whether they thought they were receiving real or sham stimulation at the end of each treatment.

### Transcranial magnetic stimulation

TMS was performed with a figure-eight coil (loop diameter 70 mm) connected to a Magstim 200^2^ stimulator (Magstim Company, Oxford, UK). The magnetic stimuli had a monophasic current waveform (rise time of 100 μs, decaying back to zero over 800 μs). The motor evoked potentials (MEPs) were registered from the right first dorsal interosseous muscle (FDI) through surface Ag/AgCl electrodes placed in a belly-tendon montage and acquired using a Biopac MP-150 electromyograph (BIOPAC Systems Inc., Santa Barbara, CA, USA), as previously reported [27].

The TMS coil was held tangentially over the scalp zone related to the primary hand motor area contralateral to the target muscle, with the coil handle pointed 45° posteriorly and laterally to the sagittal plane. The motor region was considered as the location where TMS consistently produced the largest MEP size at 120% of the resting motor threshold (rMT) in the target muscle. The region was marked with a felt tip pen on the scalp to guarantee constant placement of the coil during the whole experiment. The stimulator intensity was set to evoke a MEP approximately 1 mV peak-to-peak in the relaxed FDI at baseline, and was kept constant during the whole session. MEP amplitude measurements (average of 25 responses) were performed at baseline and at 0, 15, and 30 min after sham or real stimulation. The inter trial interval was set at 5 s (± 10%).

Throughout the experiment, complete muscle relaxation was guaranteed by audio-visual feedback where appropriate. Trials were discarded if EMG activity exceeded 100 μV in the 250 ms prior to TMS stimulus delivery. All participants were able to understand instructions, obtaining a full muscle relaxation.

### Statistical analysis

Neurophysiological parameters were compared by means of two-way repeated measures ANOVA with TIME (pre, post + 0, + 15, + 30 min) and TREATMENT (real vs. sham stimulation) as within-subjects factor. When a significant main effect was reached, post hoc tests with Bonferroni correction for multiple comparisons were conducted to analyze group-differences at respective interstimulus intervals s or time points. Mauchly’s test was used to test for assumption of sphericity, while the Greenhouse–Geisser epsilon determination was used to correct in case of sphericity violation.

Spearman’s rank-order correlation was used to assess the association between the percentage of increase in MEP amplitude and baseline rMT.

Statistical significance was assumed at *p* < 0.05. Data analyses were carried out using SPSS 21.0 software.

## Results

Regarding the differences in the subjects’ perception of the stimulation, there was no statistically significant association between the type of stimulation and its perception, as assessed by Fisher’s exact test, *p* = 1.00, suggesting that real stimulation could not be distinguished from sham stimulation.

**Fig. 2:**
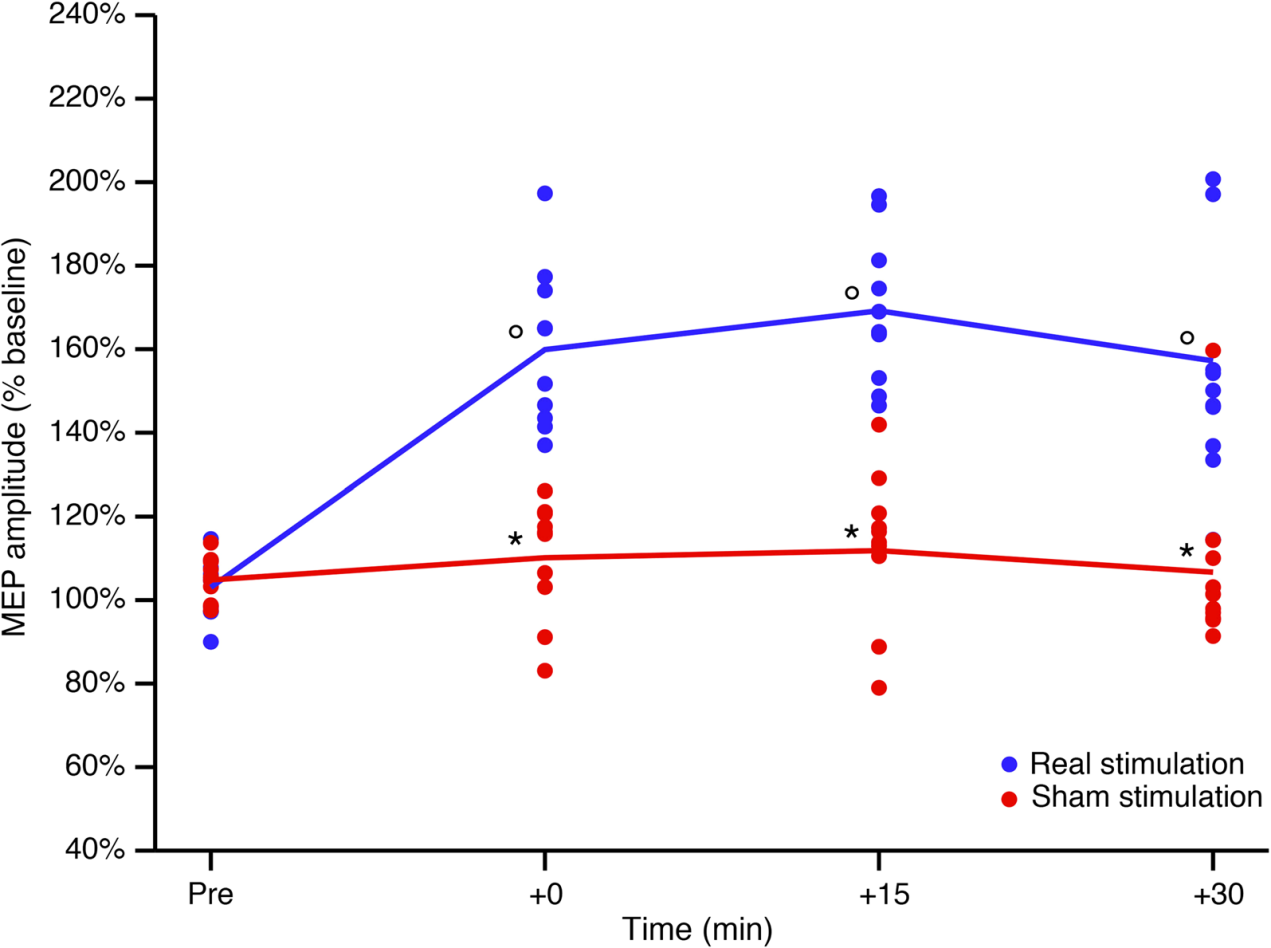
Corticospinal excitability after real and sham stimulation. Real and sham stimulation effects on corticospinal excitability, as measured by change in 1 mV MEP amplitude at various time points, in the real (blue line) and sham (red line) stimulation groups. Error bars represent standard errors. *p < 0.05 versus real stimulation. °*p* < 0.05 versus baseline (T0). *MEP* motor evoked potential, *mV* millivolt, *min* minutes

Two-way repeated measures ANOVA performed on corticospinal excitability revealed a significant TIME × TREATMENT interaction, F(3, 27) = 0.453, *p* < 0.001, partial η^2^ = 0.645. A significant main effect of TIME was observed, with significant differences in post hoc tests at + 0, + 15 and + 30 min compared to baseline after real stimulation (all *p* < 0.05) but not after sham stimulation (all *p* > 0.05). There was also a significant main effect of TREATMENT, with significant differences at + 0, + 15 and + 30 min versus baseline, for real stimulation but not for sham stimulation (all *p* < 0.005) (see Table 1, Fig. 2).

There was no significant association between the percentage of increase in average MEP amplitude and baseline rMT in both groups (real stimulation: r_s_ = 0.10, *p* = 0.776; sham stimulation: r_s_ = − 0.46, *p* = 0.177).

During the treatment phase (application of real or sham CTU Mega 20 protocol), EMG activity at high-gain amplification was monitored to highlight possible depolarizing effects, which were however absent throughout the stimulation.

## Discussion

In this study, we employed a randomized double-blind sham-controlled crossover design (to control for known and unknown factors that could potentially influenced brain activity and TMS registration) to demonstrate that Low Frequency-Pulsed Electromagnetic Fields (LF-PEMFs) induced by CTU Mega 20 were able to modulate cortical excitability in human brains, even after a single-shot application. As described above, these findings were not influenced by subject treatment expectation (real stimulation versus sham), considering its potential effect on brain activity and consequently on TMS parameters [28]. In line with transcranial static magnetic field stimulation [10, 23, 29], cortical excitability enhancement was not directly related to induced electric currents, as is the case for other neuromodulation TMS-based techniques.

In our experiment, by providing 15-min pulsed-magnetic stimulus on the primary motor area, we obtained a persistent increase of more than 60% in corticospinal excitability (as an index of Long-Term Potentiation-Like Cortical Plasticity), recording the MEP from the contralateral first dorsal interosseous muscle. This perturbation lasted for at least for 30 min after the stimulation protocol, potentially maintaining a significant difference (at least 30%), for even a longer time. As reported above, tissue biostimulation provided by CTU Meg 20 was based on non-ionizing LF-PEMFs, which could act primarily at synapse level, altering membrane ion channel function. In particular, it was shown that Ca^2+^ and Na^+^ channel activity can be perturbed by magnetic fields, considering the diamagnetic anisotropic characteristics of membrane phospholipids [8, 30, 31]. Interestingly, CTU Mega 20 is defined as a diamagnetic acceleration system, allowing high-field magnetic impulses potentially capable of inducing magnetic reorientation of membrane phospholipids and consequently inducing a biological effect on nervous system function. From this point of view, there are three points in our proof of concept study that support the idea that non-ionizing LF-PEMFs induced by the CTU Mega 20 could represent a new approach for brain neuromodulation. They are (1) CTU Mega 20 provided a larger stimulation volume (up to 27 cm^3^ compared to 1–2 cm^3^ for TMS) allowing the modulation of an extended portion of cortical surface, compared to TMS; (2) as acommercially available device, the CTU Mega 20 is ready to use, with predefined programs to perform stimulation of the nervous system, not requiring specialized staff and can be easily adapted to everyday clinical application; (3) as a completely programmable system, the CTU Mega 20 can be optimized for different types of stimulation and different neurological diseases. In conclusion, the CTU Mega 20 diamagnetic acceleration system may well be of interest in the field of neuromodulation. Further studies to optimize protocol parameters for different neurological and psychiatric conditions are warranted [32, 33].

## Conclusions

This proof-of-concept study in healthy subjects supports the idea that non-ionizing LF-PEMFs induced by the CTU Mega 20 diamagnetic acceleration system could represent a new approach for brain neuromodulation. Further studies to optimize protocol parameters for different neurological and psychiatric conditions are warranted.

## Additional files

**Additional file 1.** CONSORT 2010 checklist. CONSORT 2010 checklist of information to include when reporting a randomised trial, with the number of the page for each item reported on the right.

**Additional file 2.** CTU Mega 20^®^ technical description. Description: detailed description of the physical and technical basis of the functioning of CTU Mega 20^®^ device used in the present study.

## Abbreviations

PAS: Paired associative stimulation
LTP: Long-term potentiation
LTD: Long-term depression
LF-PEMFs: Low frequency-pulsed electromagnetic fields
Hz: Hertz
eV: Electronvolt
CTU Mega 20: Name of the device used in the present study
TMS: Transcranial magnetic stimulation
MEP: Motor evoked potential
ms: Millisecond
min: Minute
J: Joule
μs: Microsecond
FDI: First dorsal interosseous muscle
Ag/AgCl: Silver chloride
rMT: Resting motor threshold
EMG: Electromyography
μV: Microvolts
ANOVA: Analysis of variance
