# Supplementary material for: Modulation of long-term potentiation-like cortical plasticity in the healthy brain with low frequency-pulsed electromagnetic fields
Source: BMC Neurosci. 2018 Jun 13;19:34. doi: 10.1186/s12868-018-0434-z (PMC5998451; doi:10.1186/s12868-018-0434-z)
Supplement: Supplementary file 2 — Additional file 2. CTU Mega 20® technical description. Description: detailed description of the physical and technical basis of the functioning of CTU Mega 20® device used in the present study. [file 12868_2018_434_MOESM2_ESM.docx]

## **Supplementary Data: CTU Mega 20® technical description.**

## ***CTU treatments and tissue interaction.***

Before delving further into an understanding of the CTU, the equations that describe the link between a high growth rate magnetic field and tissues should be considered.

## ***Inductively Coupled effect on CTU Waveform***

The electric field induced via a time-varying magnetic field waveform is directly related to the electrical characteristics of the coil used and the current waveform applied to that coil. The induced electromotive force (EMF) is proportional to the rate of change of current in the coil (dI_coil_/dt), which produces the shape of the induced electric field [[1](#_ENREF_1)]. Coil current I_coil_(t) for an air-core inductor, driven with a voltage step V_O_, rises exponentially at the limiting current defined by the coil resistance, as:


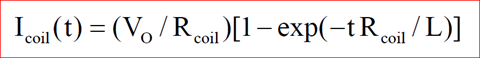


(1)

Where L is the coil inductance and R**_coil_** is the effective coil resistance, including all connecting cable and drive circuit resistances. The waveform of the induced voltage is a direct function of the time derivative of Icoil(t):


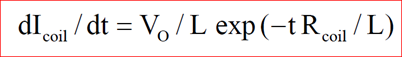


(2)

Equation 2 clearly shows that the rise time of waveform is the most important parameter related to the magnitude of effect in to the tissue [[2](#_ENREF_2)]. At same time, it is clear that very low inductance of the CTU handle coil is the key value for the optimum clinical results. Obviously, reducing the inductance by keeping the maximum B constant, the current in the coil has to be increased. There is a physical limit to the maximum drive current. Therefore, the L value is the best compromise between these two requirements in the CTU. The figure below shows the result in the time domain [[3](#_ENREF_3)].


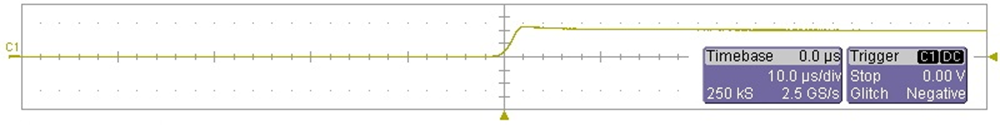


**Supplementary Figure 1:** CTU rise time at maximum power. Energy delivered to coil is about 90 Joules.

The rationale behind the first stage of CTU waveforms was based on the assumption that the induced electric field (and the associated induced current density) makes up the primary stimulus. In other words, at this stage the magnetic component was considered to be the carrier or coupler, not directly contributing to the biological effect in a significant manner. In the second stage of the CTU waveform, the assumption will be reversed. In the frequency domain, the effect is clearer [[4](#_ENREF_4)].


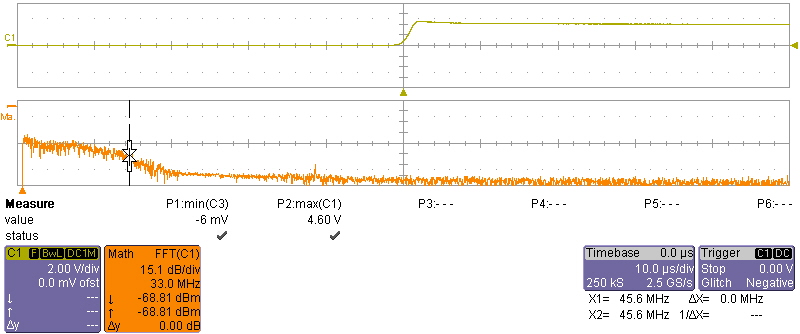


**Supplementary Figure 2:** Frequency domain of first stage of a CTU waveform.

Supplementary Figure 2 shows the spectrum related to the first stage of the CTU waveform. The total energy is released in a very high frequency form, with the spectrum knee at approximately 45MHz.

The distribution of current flow depends upon the geometry of the coil and the target. In the Finite Element Method (FEM) simulation we used and isotropic distribution of the target electrical characteristics and the real shape of the CTU coil.

The basic rule is that the voltage induced will be defined by the distribution of magnetic flux within the tissue and the electrical properties of the target [[5](#_ENREF_5)].

Since there is total symmetry in the angular (t) direction, simple electromagnetic field theory predicts that the angular component of the induced electric field, E_ɸ_, will vary, with radius r, as in:


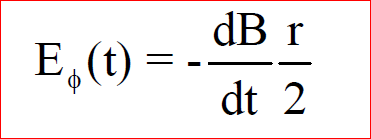


(3)

This states that the instantaneous amplitude of the induced electric field, E_ɸ_ (t), within the area of a cylindrical target, such as a laboratory dish, penetrated by a uniform magnetic field, B, is proportional to its rate of change over time, dB/dt, and the radius, r, of the target. In other words, E_ɸ_ (t) at any point in space and time is dependent upon the rate of change of B and its spatial distribution in the target [[6](#_ENREF_6)]. The actual waveform of E_ɸ_ (t) depends on dB/dt, which defines the applied frequency spectrum.

Equation 3 shows that the electric field induced in a homogenous cylindrical target is in circular loops, i.e., it is rotatory and orthogonal to the magnetic field lines. In addition, the induced E field will be greater when the magnetic field intercepts a greater cross-sectional area of the sample, i.e., the maximum E field in the target depends upon target size. The peak E field and associated current density, J, at a radius of 2 cm is often utilized for dosimetry comparisons.

Equation 3 is not valid for complex geometry and non-homogenous targets; but it is always true, from Faraday's law of induction, that the induced voltage V(t), along the line boundary of the surface S through which the flux ɸ penetrates, is:


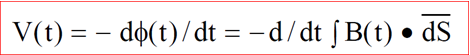


(4)

This expression indicates that, for any geometry, the time variation of induced V will also be identical to that of E in air and any other homogeneous nonmagnetic medium contained within the boundaries defined by S [[7](#_ENREF_7)].

The total energy delivered to the Body, calculated integrating the signal in Supplementary Figure 1 and equation (4) in the tissue’s physical domain included in a sphere of 0.1m radius, is about 6 Joules.

Looking into the Electrochemical Transfer Model, the same result can be reached by using the Pilla model of non-thermal, sub-threshold electromagnetic fields [[8](#_ENREF_8)]. Ionic interactions at electrically charged interfaces of a cell are generally voltage dependent (electrochemical) processes. Several distinct types of electrochemical interactions can occur at cell surfaces. One includes all of the non-specific electrostatic interactions involving water dipoles and hydrated (or partially hydrated) ions at the lipid bilayer/aqueous interface of a cell membrane, which makes all cell membranes capacitors. A second interaction involves voltage dependent ion/ligand binding. Here an ion or dipole can effectively compete with water dipoles and hydrated ions for specific membrane sites, which, in turn can modulate a downstream cascade.

An equivalent electrical circuit model representing these electrochemical processes at cell surfaces and junctions, related to the current i_b_(s), which flows into this pathway, can be written:


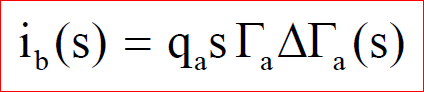
 (5)

Where q_a_ is a coefficient representing the dependence of interfacial charge upon the surface concentration of the bound ion, Γ_a_, and s (=σ+jω) is the complex frequency variable of the Laplace transform [[9](#_ENREF_9)].

Equation 5 shows that the current in this pathway is a function of the change in surface concentration of the binding ion with time, ΔΓ_a_(s), which, in turn, is voltage dependent and a function of the change in the surface concentration of the product of the follow-up biochemical reaction, ∆β_b_(s). In order to derive an expression for the impedance, Z_A_(s), of this pathway, it is necessary to define relationships between ∆Γ_a_ and the change in transmembrane voltage (V_M_), and ∆β_b_(s). This may be written, for first order linear kinetics, as:


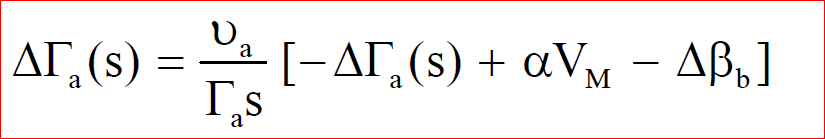
 (6)

where υ_a_ is the binding rate constant and α is proportional to the potential dependence of binding (≅∂Γ/∂V_M_). The change in surface concentrations of the ion and the biochemical product can also be described by first order kinetics:


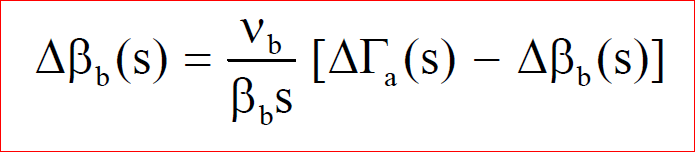
 (7)

Where v_b_ is the rate constant for the follow up chemical reaction (defined as for v_a_) governing the rate of formation (decomposition) of the bound biochemical product after ion binding in a molecular cleft has occurred [[10](#_ENREF_10)].

The equations above allow the electrical impedance of the proposed transduction pathway at the cell membrane, Z_A_(s), to be written as:


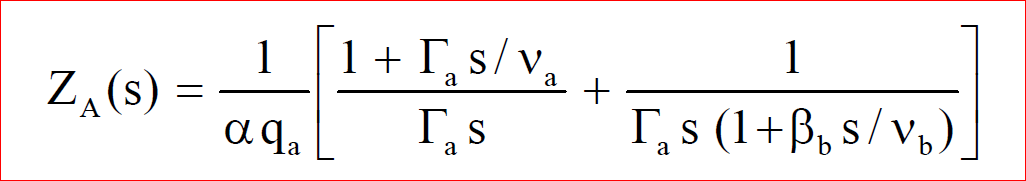
 (8)

Equation 9 reveals the existence of two time constants, the parameters of which are identifiable in terms of the rate constant and change in surface concentration of each reaction step.

An electrical equivalent circuit, Z_A_ is below [[11](#_ENREF_11)].

R**A**


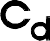

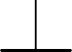

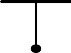

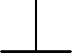

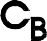

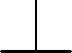

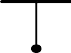

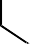

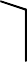

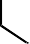

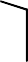

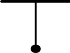

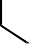

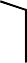

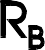

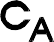


Fig3: Electrical equivalent model.

In order to increase the current in Z_A_ we have to increase the signal bandwidth and the maximum frequency below the CTU spectrum waveform related to the first stage where there is the fastest transition [[12](#_ENREF_12)].


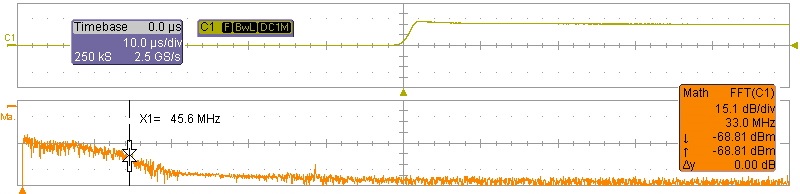


**Supplementary Figure 4:** Maximum CTU Frequency emission.

## ***Magnetic field and Larmor Precession effect on CTU Waveforms***

A bound ionic oscillator in a static magnetic field will precess at the Larmor frequency in the plane perpendicular to the applied field. This motion will persist in superposition with thermal forces, until the thermal forces eventually eject the oscillator from a binding site. For a magnetic field oriented along the z-axis, the precessional motion will be confined to the x-y plane. The Larmor Processor Model (LPM) proposes that the biochemical reactivity of a bound ion complex may be affected by changes in the spatial orientation of the bound ionic oscillator [[13](#_ENREF_13)].

The effect of magnetic fields according to the LPM can be summarized by the Lorentz-Langevin equation written to describe the motion of an ion bound in a potential well (molecular cleft) subject to a magnetic field oriented along the z-axis in the presence of thermal noise forces is:


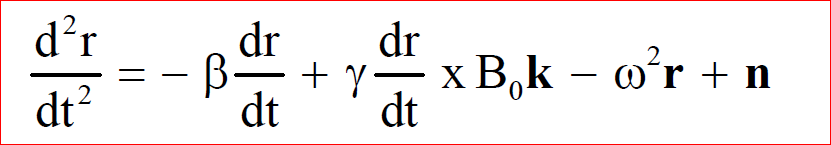
 (10)

Where: r is the position vector of the ion; β is the viscous damping coefficient per unit mass (due to molecular collisions in the thermal bath), γ is the ion charge to mass ratio; B_0_ is the magnitude of the magnetic field vector; k is the unit vector along the z-axis; ω is the angular frequency of the oscillator and n is the random thermal noise force per unit mass.

Equation 10 describes the motion of an oscillator (ion) in a molecular cleft due to an exogenous magnetic field in the presence of thermal noise [[14](#_ENREF_14)]. The solution may be written:


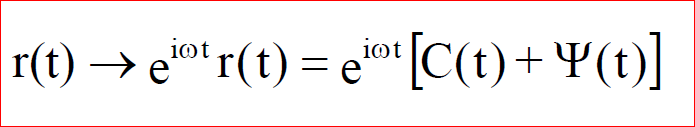
 (11)

Where r(t) is the position vector of the bound ion; C(t) is the coherent oscillation of the bound oscillator and Ψ(t) is the contribution due to thermal noise forces. The ion trajectory thus consists of a coherent part, given by:


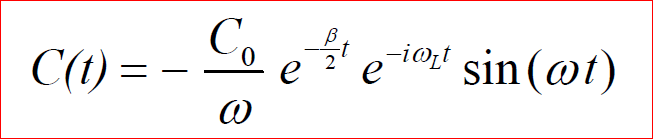
 (12)

Where C_0_ is determined by initial conditions and ωL (= γB/2) is the Larmor frequency and a component due to thermal noise:


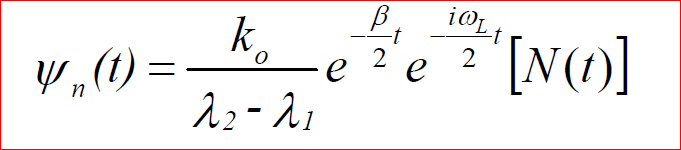
 (13)

Where k_o_ is determined by initial conditions; N(t) is the accumulation of the thermal component over time; and λ_1_ and λ_2_ are the roots and contain the AC and DC magnetic field terms.

The thermal component Ψ(t) of the ion trajectory itself thus consists of a harmonic oscillator driven by thermal noise, subject to viscous damping and undergoing precessional motion at the Larmor frequency about the axis defined by the magnetic field. It oscillates at the fundamental frequency of the oscillator potential with amplitude increasing over time, ultimately resulting in ejection from the binding site after a bound lifetime determined by the magnitude of thermal forces [[15](#_ENREF_15)].

Both the coherent and thermal components of an ion at a binding site exhibit Larmor precession in the presence of an applied magnetic field.

In order to have many different RF frequency emissions from the CTU, B_o_ must be changed, assuming many values as slowly as possible.

Larmor precession converts the exogenous magnetic field amplitude into a frequency determined by the gyromagnetic ratio of the target. Thus, for an ion oscillating along the z axis the Larmor frequency ω_L_ is [[16](#_ENREF_16)]:


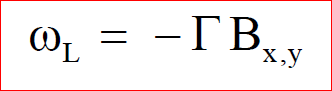
 (14)

Where Γ = q/2m for a simple unhydrated ion.


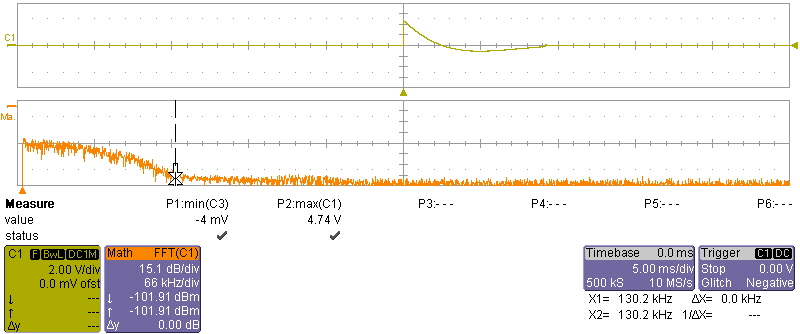


**Supplementary Figure 5:** signal in stage 2 of CTU Waveform.

The shape in the stage 2 of the CTU waveform is very different to stage 1 described in the previous paragraph.

Here the Magnetic field decays almost linearly from the maximum value to 0 assuming any intermediate value of B_o_. At this stage, the bandwidth of spectrum is not relevant because equation 11 is dominant with respect to equation 3.

Using a linear decay according to equation 14, it is certain that any Larmor frequencies will have almost the same time effect [[17](#_ENREF_17)].

It is difficult to correctly simulate the energy delivered to the tissue at this stage. However, assuming a COP of 0.1 as the Larmor conversion and integrating the signal in Supplementary Figure 5 in the same sphere of 0.1 m radius about 3 J of energy will be had.

***Conclusion.***

CTU Waveform is designed to maximize the conversion rate from a magnetic field to electrical energy for span of many Larmor frequencies. This design minimizes the cell depolarization effect and does not effect the electrodynamic and diamagnetic force responsible for the CTU goal.

**REFERENCES**

1. Weintraub M: Magnetic bio-stimulation in painful diabetic peripheral neuropathy: a novel intervention-a randomized, double-placebo crossover study. Am J Pain Manage 1999, 9:8-17.

2. Binhi VN: Amplitude and frequency dissociation spectra of ion-protein complexes rotating in magnetic fields. Bioelectromagnetics 2000, 21(1):34-45.

3. Chiabrera A, Bianco B, Moggia E, Kaufman JJ: Zeeman-Stark modeling of the RF EMF interaction with ligand binding. Bioelectromagnetics 2000, 21(4):312-324.

4. Wolsko PM, Eisenberg DM, Simon LS, Davis RB, Walleczek J, Mayo-Smith M, Kaptchuk TJ, Phillips RS: Double-blind placebo-controlled trial of static magnets for the treatment of osteoarthritis of the knee: results of a pilot study. Altern Ther Health Med 2004, 10(2):36-43.

5. Buechler DN, Christensen DA, Durney CH, Simon B: Calculation of electric fields induced in the human knee by a coil applicator. Bioelectromagnetics 2001, 22(4):224-231.

6. Zborowski M, Midura RJ, Wolfman A, Patterson T, Ibiwoye M, Sakai Y, Grabiner M: Magnetic field visualization in applications to pulsed electromagnetic field stimulation of tissues. Ann Biomed Eng 2003, 31(2):195-206.

7. Colbert AP MM, Banerij M, Pilla AA: Magnetic mattress mad use in patients with fibromyalgia: A randomized double-blind pilot study. J Back Musculoskeletal Rehab 1999, 13(1):19-31.

8. Alfano AP, Taylor AG, Foresman PA, Dunkl PR, McConnell GG, Conaway MR, Gillies GT: Static magnetic fields for treatment of fibromyalgia: a randomized controlled trial. J Altern Complement Med 2001, 7(1):53-64.

9. Weintraub MI, Wolfe GI, Barohn RA, Cole SP, Parry GJ, Hayat G, Cohen JA, Page JC, Bromberg MB, Schwartz SL: Static magnetic field therapy for symptomatic diabetic neuropathy: a randomized, double-blind, placebo-controlled trial. Arch Phys Med Rehabil 2003, 84(5):736-746.

10. Brown CS, Ling FW, Wan JY, Pilla AA: Efficacy of static magnetic field therapy in chronic pelvic pain: a double-blind pilot study. Am J Obstet Gynecol 2002, 187(6):1581-1587.

11. Sisken BF, Kanje M, Lundborg G, Kurtz W: Pulsed electromagnetic fields stimulate nerve regeneration in vitro and in vivo. Restor Neurol Neurosci 1990, 1(3):303-309.

12. Greenebaum B, Sutton CH, Vadula MS, Battocletti JH, Swiontek T, DeKeyser J, Sisken BF: Effects of pulsed magnetic fields on neurite outgrowth from chick embryo dorsal root ganglia. Bioelectromagnetics 1996, 17(4):293-302.

13. Man D, Man B, Plosker H: The influence of permanent magnetic field therapy on wound healing in suction lipectomy patients: a double-blind study. Plast Reconstr Surg 1999, 104(7):2261-2266; discussion 2267-2268.

14. Muehsam DJ PA: Weak magnetic field modulation of ion dynamics in a potential well: mechanistic and thermal noise considerations. Bioelectrochem Bioenerg 1994, 35:71-79.

15. Pilla AA KJ, Ryaby JT.: Electrochemical kinetics at the cell membrane: A physicochemical link for electromagnetic bioeffects. In: *Mechanistic Approaches to Interactions of Electric and Electromagnetic Fields with Living Systems.* edn. Edited by Findl MBaE. New York, USA: Springer US; 1987: 39-62.

16. Barnes FG, B.: Handbook of Biological Effects of Electromagnetic Fields. In*.*, 3rd edn.: CRC Press; 2006.

17. Zhadin MN: Combined action of static and alternating magnetic fields on ion motion in a macromolecule: theoretical aspects. Bioelectromagnetics 1998, 19(5):279-292.
